# Supplementary material for: Non-Invasive Monitoring of Increased Fibrotic Tissue and Hyaluronan Deposition in the Tumor Microenvironment in the Advanced Stages of Pancreatic Ductal Adenocarcinoma
Source: Cancers (Basel). 2022 Feb 16;14(4):999. doi: 10.3390/cancers14040999 (PMC8870395; doi:10.3390/cancers14040999)
Supplement: Supplementary file 1 [file cancers-14-00999-s001.zip › cancers-1546934-Suppl-XML - update.pdf]

cancers

MDPI

Supplementary Materials: Non-Invasive Monitoring of Increased Fibrotic Tissue and Hyaluronan Deposition in the Tumor Microenvironment in the Advanced Stages of Pancreatic Ductal Adenocarcinoma

Ravneet Vohra, Yak-Nam Wang, Helena Son, Stephanie Totten, Akshit Arora, Adam Maxwell and Donghoon Lee

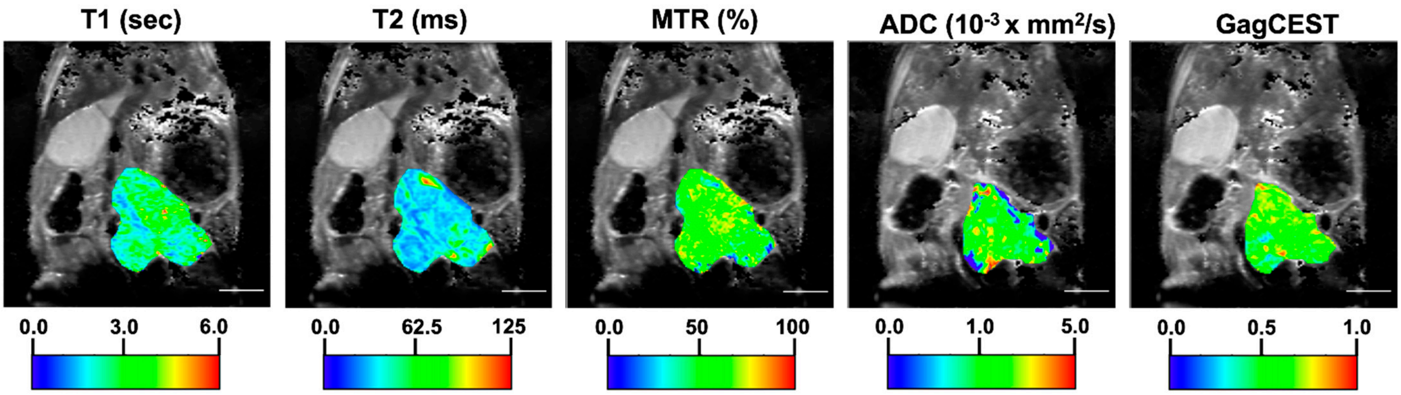

Figure S1. Representative colored maps with T1, T2, MTR, ADC and GagCEST measures of pancreatic tumor from a KPC mouse. Scale bar = 5 mm.

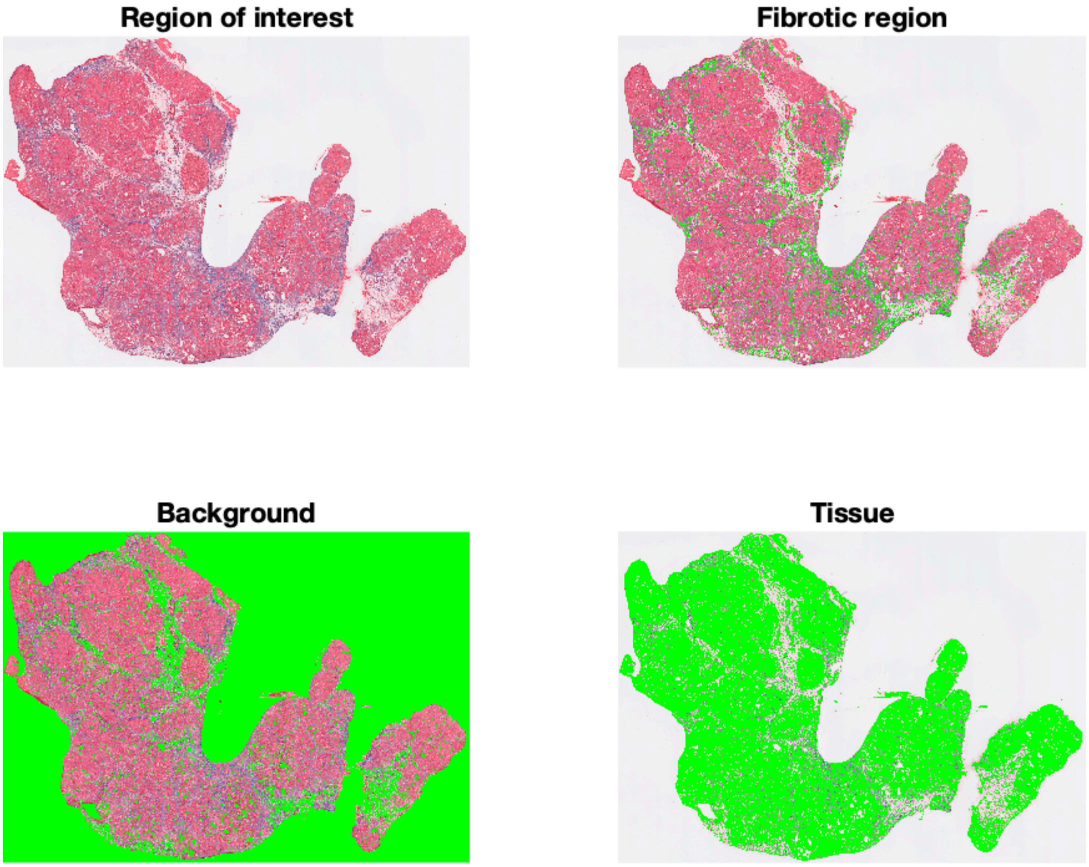

Figure S2. Representative image of methodology demonstrating the technique used to quantify connective tissue deposition in the region of interest in the pancreatic tumor of KPC mouse.
